# 伴IRF2BP2-RARA融合基因的变异型急性早幼粒细胞白血病1例报告并文献复习

**DOI:** 10.3760/cma.j.issn.0253-2727.2023.03.013

**Published:** 2023-03

**Authors:** 成业 邬, 世伟 杨, 玉龙 李, 晓燕 董, 润红 于, 琳 张, 保军 商, 平玲 史, 尊民 朱

**Affiliations:** 1 河南省人民医院血液病研究所，河南省血液病理重点实验室，河南省CAR-T细胞治疗与转化工程研究中心，河南省干细胞分化与调控重点实验室，郑州 450003 Institute of Hematology of Henan Provincial People's Hospital, Henan Key Laboratory of Hematopathology, Henan Provincial Engineering Research Center of CAR-T Cell Treatment and Transformation, Henan Key Laboratory of Stem Cell Differentiation and Modification, Zhengzhou 450003, China; 2 河南省眼科研究所，河南省人民医院，郑州 450003 Henan Eye Institute, Henan Provincial People's Hospital, Zhengzhou 450003, China

急性早幼粒细胞白血病（APL）是以形态异常的早幼粒细胞、凝血障碍、特异性染色体异常、对维甲酸（ARTA）治疗有独特反应为特征的急性髓系白血病（AML）亚型。大多数APL患者携带t（15;17）（q22;q12），从而导致早幼粒细胞白血病基因（PML）与RARA融合[1]–[4]。PML-RARA融合基因既是分子病因又是治疗靶点[5]–[6]。除了最常见的PML-RARA融合基因的存在，近年来一些新的融合基因不断被发现[7]–[10]。最近，我们发现1例变异型APL患者RARA罕见的伙伴基因IRF2BP2，由IRF2BP2外显子1和RARA外显子3断裂拼接，重新形成IRF2BP2-RARA融合基因。这是国内报道的第1例伴有IRF2BP2-RARA融合基因的变异型APL，现报道如下并进行文献复习。

## 病例资料

患者，男性，60岁，因头晕乏力、鼻腔出血入院。查体：轻度贫血面容，全身皮肤黏膜无黄染，无皮疹、皮下出血、皮下结节、瘢痕，毛发分布正常，皮下无水肿，无肝掌、蜘蛛痣。口唇无发绀，口腔黏膜正常，舌苔正常。血常规：WBC 5.92×10^9^/L、RBC 2.86×10^12^/L、HGB 97.0 g/L、PLT 19×10^9^/L。凝血功能：血浆凝血酶原时间（PT）13.5 s（正常参考范围11～17 s），活化部分凝血活酶时间（APTT）31.4 s（正常参考范围28.0～43.5 s），纤维蛋白原（FIB）3.11 g/L（正常参考范围2～4 g/L），D-二聚体36.83 mg/L（正常参考范围≤0.5 mg/L）。外周血涂片分类计数：外周血幼稚细胞占77％。髂骨穿刺骨髓涂片分类：①骨髓增生极度活跃，粒系异常增生，以颗粒增多的异常早幼粒细胞为主，占0.928，其胞体大小不等，呈圆形、椭圆形或泪滴形；核圆形、类圆形或不规则形，易见凹陷、折叠等；染色质细致，核仁2～4个，隐显不一；胞质量中等或丰富，着蓝色，含有大量细小的嗜天青颗粒，易见内外浆现象，可见Auer小体。中幼粒及以下阶段细胞少见或缺如。②红系增生受抑制。成熟红细胞大小不等，色素充盈尚可。③淋巴细胞可见。④全片未见巨核细胞，血小板少见，形态无明显异常。细胞化学染色：POX阳性率100％。免疫分型显示异常髓系幼稚细胞占有核细胞的91.24％，表达CD117、CD33、CD13、CD123、CD64、MPO，部分表达CD56、CD15，不表达CD34、CD38、HLA-DR、CD11b、CD4、CD300e、CD10、CD19、CD20、CD4、CD8、CD3、CD7、CD2、cCD79a、cCD3、CD22、CD5、cTDT。染色体核型：46，XY[20]；荧光原位杂交：RARA基因断裂重排。白血病相关基因突变检测发现患者存在KIT（D816V）、ARID1B（G1704X）、WT1（A382fs）突变；荧光定量PCR结果显示白血病相关56种融合基因筛查均阴性，其中包括PML-RARα（L型）、PML-RARα（S型）、PML-RARα（V型）、PLZF-RARα、NPM-RARα、STAT5b-RARα、FIP1L1-RARα、PRKAR1A-RARα、NUMA1-RARα等已报道的较常见的RARA重排伙伴基因。mRNA测序结果示1号染色体与17号染色体易位形成IRF2BP2-RARA融合基因，一代测序证实断点位于IRF2BP2 cDNA的1031位（NCBI参考序列：NM_001077397.1）和RARA cDNA的648位（NCBI参考序列：NM_000964.4），对比数据库发现IRF2BP2-RARA融合涉及到IRF2BP2基因的1号外显子和RARA基因的3号外显子（图1）。诊断：变异型APL。予亚砷酸（ATO）+ ATRA双诱导治疗。治疗过程中患者出现重症感染，多脏器功能衰竭，抢救无效死亡，总生存期仅14 d。

**图1:**
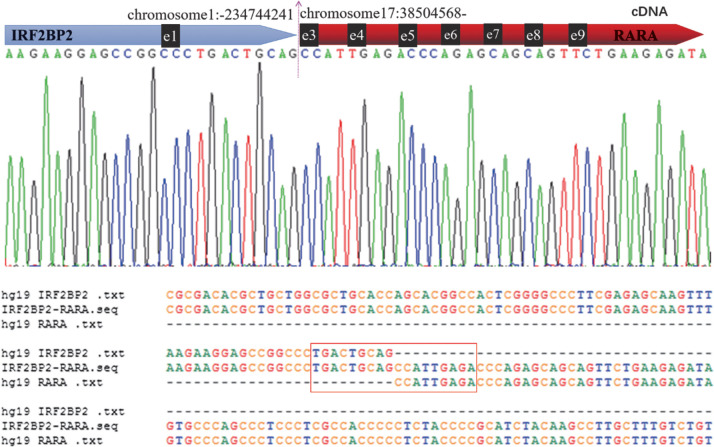
变异型急性早幼粒细胞白血病患者IRF2BP2与RARA融合测序结果与序列比对

## 讨论及文献复习

位于染色体1q42.3的IRF2BP2基因编码的IRF2BP2蛋白包含一个N端锌指和一个C端环指结构域，该结构域与IRF2.1的C端转录抑制结构域特异性相互作用；IRF2BP2蛋白作为一种转录共抑制因子，抑制活化T细胞的核因子的激活，参与调控细胞周期、分化和凋亡相关基因的表达。IRF2BP2蛋白的直接靶基因是TP53，其过度表达通过抑制TP53介导的TP21和BAX基因的转录来抑制凋亡[11]。在乳腺癌细胞系中，IRF2BP2蛋白被鉴定为抗凋亡因子，在意义未明的单克隆丙种球蛋白病中被鉴定为肿瘤相关抗原[12]。

RARA通过与维甲酸反应元件结合作为一种异二聚体与维甲酸X受体（RXR）发挥作用，这种RARA-RXR复合物是早幼粒细胞分化所必需的。不同的X-RARA融合蛋白可能对RARA-RXR复合物具有显著的负调节作用，或形成将RXR隔离的异二聚体，并招募协同抑制因子和组蛋白去乙酰基酶复合物来抑制与骨髓分化相关基因的表达。同样，IRF2BP2-RARA融合蛋白可能作为一种RARA和IRF2BP2的显著负调节因子促进白血病的发生[13]。

ATRA通过靶向作用于PML-RARA蛋白的RARA部分诱导肿瘤细胞分化，ATO通过靶向作用于PML-RARA蛋白的PML部分促进肿瘤细胞凋亡[2],[14]–[15]。本文报道的融合基因可能导致IRF2BP2蛋白功能异常和细胞分化凋亡受损，同时RARA基因异常也导致造血细胞分化停滞在早幼粒阶段。在该病例中，患者因为特殊的基因融合形式导致了RARA蛋白的部分保守结构域的丢失，从而对ATRA不敏感[16]。而RARA的伙伴基因是IRF2BP2而非PML，使患者因无ATO结合位点而对其无效，这些和患者使用ATRA和ATO治疗临床疗效欠佳是一致的，这可能就是ATRA和ATO对部分伴IRF2BP2-RARA融合基因APL治疗效果不佳的分子机制。

AML相关基因检测发现本例患者同时存在KIT、WT1和ARID1B基因突变。KIT编码蛋白属于受体酪氨酸激酶，参与包括造血干细胞在内的多种细胞的发育过程；WT1突变见于15％的AML，过表达或基因突变常参与AML和急性淋巴细胞白血病的发生，KIT和WT1基因突变与预后不良相关联[17]–[18]。ARID1B基因编码SWI/SNF蛋白复合物的一个亚基，SWI/SNF复合物通过称为染色质重塑的过程调节基因表达。该基因过表达可引起ATRA耐药，突变阳性者预后不良，复发风险高[19]。同时本例患者在治疗过程中出现严重感染，无法继续化疗，所有上述分子生物学特征及患者临床状况可能也是本例患者治疗效果差，在未取得缓解情况下短期内死亡的原因。

武妮等[20]研究表明，除初诊时变异型APL患者PLT高于经典型APL外，两种亚型在其他方面的差异均无统计学意义。本例IRF2BP2-RARA融合基因的变异型APL患者为国内首例、国际第7例报道。该7例患者临床实验室特征详见表1[3],[11],[21]–[24]，IRF2BP2基因与RARA基因融合形式多样，2例染色体核型分析发现1号和17号染色体易位，3例患者有出血症状，6例患者能够取得完全缓解（CR），但其中4例1年内复发或死亡，总体预后欠佳。

**表1:** 已报道7例伴IRF2BP2-RARA融合基因急性早幼粒细胞白血病（APL）患者临床及实验室特征、治疗方案与转归

参考文献	性别	年龄（岁）	血常规			染色体核型	IRF2BP2-RARA断裂融合形式	出血	治疗方案	转归
			WBC（×10^9^/L）	HGB（g/L）	PLT（×10^9^/L）					
Yin等[3]	女	19	4.5	91	29	正常核型	IRF2BP2 exon 2融合RARA intron 2	瘀斑、鼻出血	ATRA、ATO、吉妥珠单抗、奥佐米星、伊达比星	CR后10个月复发
Liu等[11]	女	32	5.1	64	33	45,X,–X	IRF2BP2 intron 1融合RARA intron 2	月经过多	ATRA、ATO、蒽环类药物、Ara-C、高三尖杉酯碱	CR后12个月复发死亡
Shimomura等[21]	女	68	1.65	82	48	–X	IRF2BP2 exon1 融合 RARA exon 3-9	未报道	ATRA、阿糖胞苷、伊达比星、吉妥珠单抗、奥佐米星	CR后12个月复发，确诊后27个月死亡
Jovanovic等[22]	男	37	3.8	125	7	正常核型	IRF2BP2 intron 1融合RARA intron 2	未报道	ATRA、蒽环类单药化疗	CR大于50个月
Mazharuddin等[23]	男	34	4.1	93	23	45,X,–Y,t(1;17)(q42;q21),i(8)(q19)	IRF2BP2 exon1融合 RARA exon 3	未报道	ATRA、伊达比星、阿糖胞苷	CR大于18个月
Alotaibi等[24]	女	76	10.5	70	34	45,X,–X,der(1),t(1;17)(q42;?),t(2;17)(p24;q21),del(10)(q22)	IFR2BP2 exon 2融合RARA exon 3	未报道	ATRA、ATO、伊达比星	CR后2周复发死亡
本例	男	60	5.9	97	19	正常核型	IFR2BP2 exon 1融合RARA exon 3	鼻腔间断出血	ATRA、ATO、柔红霉素	确诊后2周死亡

**注** ATRA：全反式维甲酸；ATO：亚砷酸；Ara-C：阿糖胞苷；CR：完全缓解

本病例从初诊时骨髓涂片细胞学特征、髓过氧化物酶反应性考虑APL，但染色体正常核型及常见RARA基因重排阴性让诊断无法明确，最终通过荧光原位杂交技术及RNAseq检测RARA基因重排明确了变异型APL的诊断。这是国内首次在骨髓细胞形态和免疫表型上与APL相似的患者中发现IRF2BP2参与变异型APL的病例报告。因此，我们建议对于细胞遗传学或常见融合基因分子生物学阴性，骨髓细胞形态、流式免疫分型高度怀疑APL的患者，系统地使用测序分析或RNAseq或许能够得到明确的诊断。本研究鉴定出IRF2BP2-RARA融合基因，我们报告的1例患者临床治疗效果较差，国外报道的6例伴IRF2BP2-RARA融合基因的变异型APL患者在取得CR后4例短期复发或死亡，表明伴该类融合基因的APL可能显示出不良的预后。但鉴于目前病例数较少，因此尚需进行更多的临床研究以确定伴IRF2BP2-RARA变异型APL远期疗效及预后情况。
